# Association Between a Technology-Enabled Postpartum Program and Cardiovascular Disease Risk Reduction in Women With Prior Hypertensive Disorders of Pregnancy

**DOI:** 10.1097/og9.0000000000000087

**Published:** 2025-06-05

**Authors:** Dan Yedu Quansah, Natalie Martin, Lisa McDonnell, Kara Nerenberg, Robert D. Reid, Andrew L. Pipe, Thais Coutinho, Hassan Mir, Graeme Smith, Jessica Pudwell, Kerri-Anne Mullen

**Affiliations:** Canadian Women Heart Health Centre, University of Ottawa Heart Institute, the Division of Cardiac Prevention and Rehabilitation, University of Ottawa Heart Institute, the Champlain Regional Stroke Network, the Ottawa Hospital, and the Faculty of Medicine, University of Ottawa, Ottawa, the Department of Obstetrics & Gynecology, Queen's University, Kingston, Ontario, and the Departments of Medicine, Obstetrics & Gynecology, and Community Health Sciences, University of Calgary, Calgary, Alberta, Canada; and the Department of Cardiovascular Medicine, Mayo Clinic, Rochester, Minnesota.

## Abstract

**BACKGROUND:**

To test the association between a behavior-change, risk-reduction intervention and cardiovascular disease (CVD) risk scores in women with previous hypertensive disorders of pregnancy (HDP).

**METHODS:**

We completed a prospective, single-arm comparative pre–post study of women with recent HDP. The intervention was the CardioPrevent Postpartum Program, which involves health behavior-change coaches who counseled participants in 17 sessions over 12 months regarding behavioral changes (smoking, nutrition, physical activity), and worked with primary-care practitioners to optimize medical management of underlying risk factors (hypertension, dyslipidemia, diabetes mellitus). We measured changes in global risk scores (lifetime, Framingham Risk score) and clinical, psychosocial, and health behavior outcomes at 12 months compared with baseline.

**RESULTS:**

Overall, 190 women were enrolled. Absolute reductions in Framingham and Lifetime Risk Scores at 12 months postintervention were 53.7% and 22.6%, respectively (both *P*≤.001). The intervention was associated with decreases in body weight (81.7±19.4 vs 74.1±19.8 kg, *P*<.001), waist circumference (96.9±15.5 vs 89.5±14.9 cm, *P*<.001), hypertension (50% vs 4.3%), and metabolic syndrome (53.6% vs 43.5%) between baseline and 12 months, but fasting plasma glucose and hemoglobin A_1c_ were unchanged. Triglycerides, low-density lipoprotein cholesterol, and total cholesterol decreased; however, high-density lipoprotein cholesterol also decreased. We observed a 40.6% absolute reduction in postpartum depression, a 28.2% absolute reduction in anxiety, and a 67.9% absolute reduction in perceived stress at 12 months compared with baseline. Compared with baseline, the intervention was associated with a higher proportion of participants who met guideline recommendations for physical activity levels (48.4% vs 75.2%, *P*<.001), fruit and vegetable intake (21.5% vs 44.4%, *P*<.001), and medication adherence (29.4% vs 48.7%, *P*<.001) at 12 months.

**CONCLUSION:**

This behavior-change, risk-reduction intervention was associated with improvements in CVD risk factors at 12 months after initiation. Multidisciplinary health behavior-change programs may be effective CVD risk-reduction strategies for women with previous HDP.

## Abstract 2

A behavior-change, risk-reduction intervention was associated with improvements in cardiovascular disease risk factors among women with previous hypertensive disorders of pregnancy.

Cardiovascular disease (CVD) is the leading cause of death among women globally.^1^ Overall, women experience poorer outcomes compared with men after a cardiovascular event, and myocardial infarctions account for one-third of global female deaths.^2^ Compared with men, several traditional risk factors for CVD pose a greater risk for women such as smoking, hypertension, diabetes mellitus, obesity, physical inactivity, depression, and anxiety.^3^ In addition, women may experience sex-unique CVD risk factors such as hypertensive disorders of pregnancy (HDP), gestational diabetes mellitus, polycystic ovarian syndrome, and menopause. These sex-specific risk factors increase the risk of CVD in women twofold to fourfold.^4^

Changes in health behaviors, such as quitting smoking, enhancing diet quality, increasing physical activity, and reducing alcohol consumption can decrease CVD risk in women. Cardiovascular society guidelines reiterate the need for primary prevention of CVD (including risk-factor management), and awareness and educational programs to improve CVD outcomes. In women with prior HDP, lifestyle changes after pregnancy have been shown to be an effective strategy to prevent future CVD.^5^ Studies suggest that lifestyle counseling programs such as health behavior-change coaching strategies for individuals at risk of CVD typically result in CVD risk factor improvement^6^ and can be adopted for women with previous HDP early in the postpartum period.

Although the postpartum period provides a window of opportunity to improve risk, recovery and childcare mean that this period can be intense and challenging. Furthermore, it is rare that health care practitioners can effectively personalize and deliver lifestyle counseling given the constraints surrounding patient–clinician interactions during this time. Technology-enabled health behavioral counseling approaches that address education, awareness, and behavior change could maximize intervention effectiveness and adherence in the postpartum period by reducing face-to-face appointments while increasing patient interaction virtually.^7^ This study evaluated the association between a technology-enabled behavior-change, risk-reduction program and both CVD risk scores and individual risk-factor changes in women with previous HDP in the postpartum period.

## METHODS

We completed a single-arm comparative pre–post study of the CardioPrevent Postpartum Program (CP-PP). Women eligible for inclusion in this analysis were those 18 years or older who had a pregnancy complicated by preeclampsia with or without severe features, who were within 25 weeks postpartum (as determined by diagnosis on the referral from an obstetrician, family physician, or nurse practitioner), and were enrolled in the CP-PP at the University of Ottawa Heart Institute between July 2013 and August 2022. We excluded individuals previously diagnosed with CVD, those diagnosed with HDP for longer than 5 years, and those who had new pregnancies at any time during the 12-month program. The study protocol was approved by the Ottawa Health Science Network Research Ethics Board (Protocol 20220117-01H).

The CP-PP is a global CVD evidence-based, tailored primary prevention program for women who experienced a recent (less than 5 years) adverse pregnancy condition, including HDP. The CP-PP was adapted from the CardioPrevent program (previously described),^8^ developed at the University of Ottawa Heart Institute (http://pwc.ottawaheart.ca/care/) to reduce global CVD risk in a general population of patients with at least 10% predicted 10-year risk of an adverse CVD outcome.^8^ Briefly, CardioPrevent is based on cognitive-behavioral strategies, which are essential components of behavior change. The focus is on modifying how individuals think about themselves, their behaviors, and their surrounding circumstances, as well as how to adjust their lifestyles. Additionally, the frequent and prolonged timing of the sessions supports success across sequential stages of behavior change.

**Fig. 1.**
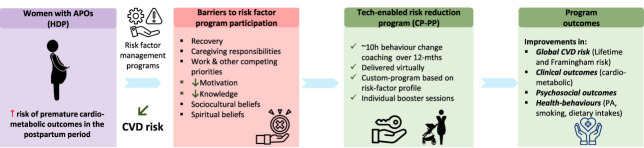
Program description and outcomes of the behavior-change, risk-reduction intervention. APO, adverse pregnancy outcomes; CP-PP, CardioPrevent Postpartum Program; CVD, cardiovascular disease; HDP, hypertensive disorders of pregnancy; mths, months; PA, physical activity.

The CP-PP is a technology-enabled intervention primarily delivered virtually by trained behavior-change coaches who assist patients with smoking cessation, dietary modification, and physical activity to improve CVD risk reduction. Participants in the CP-PP receive approximately 10 hours of health coaching over 12 months using a customized program plan based on participant risk-factor profile. When a participant is referred to the program by a physician or nurse practitioner and screened for eligibility, an intake visit is scheduled for baseline assessment. The program consists of up to 17 coaching sessions. Session 1 is a face-to-face meeting between the participant and their coach to review their baseline risk factor profile (eg, diabetes, blood pressure, blood pressure medication, smoking, physical activity levels, family history of CVD, and CVD risk factors) and to individualize the program. Sessions 2 to 11 are behavioral-counseling sessions delivered by telephone over a period of 6 months. At 6-month follow-up (session 12), baseline measures are repeated, and the program is further tailored to the participant's needs. Sessions 13 to 16 are individual behavior-change counseling booster sessions to further improve outcomes. The final session (session 17) is at 12-month follow-up where outcomes are reassessed. Figure 1 provides an overview and timeline of the CP-PP.

*Quansah. Effectiveness of Postpartum Program on CVD Risk Scores. O&G Open 2025*.

All outcomes were assessed at baseline, 6 months, and 12 months. Primary outcomes were global CVD risk scores. Secondary outcomes included clinical, psychosocial, and health-behavior outcomes. We assessed global CVD risk using Lifetime Risk Score and Framingham Risk Score, both measured as a percentage. Lifetime Risk Score takes into account age, sex, race, lipid levels, blood pressure, medication use, diabetes status, and smoking status in estimating the CVD risk score.^9^ A score less than 39% represents a low risk; 39% to 50% represents an intermediate risk, and 50% or greater is considered a high risk. The Framingham Risk Score is calculated based on the following risk factors: sex, age, total cholesterol, high-density lipoprotein (HDL) cholesterol, systolic blood pressure, and cigarette use.^10^ Risk of CVD is considered low if the Framingham Risk Score is less than 10%, moderate if between 10% and 19%, and high if 20% or greater.

Clinical outcomes included weight, body mass index (BMI, calculated as weight in kilograms divided by height in meters squared), and waist circumference (measured in cm at the level of the umbilicus); these were measured using standard procedures performed by a trained health coach. We also assessed blood pressure, total cholesterol, HDL, low-density lipoprotein (LDL), total cholesterol/HDL ratio, triglycerides, fasting plasma glucose (FPG), hemoglobin A_1c_, and metabolic syndrome. *Metabolic syndrome* was defined according to the International Diabetes Federation guidelines, which is based on either a waist circumference (metabolic syndrome – waist circumference) greater than 80 cm or BMI of 30 or higher (metabolic syndrome – BMI) and at least two of the following cutoffs: triglycerides 1.7 mmol/L or higher, HDL less than 1.3 mmol/L, blood pressure 130/85 mm Hg or higher, FPG 5.6 mmol/L or higher, or type 2 diabetes.

Psychosocial outcomes included anxiety, depression, perceived stress, and mental and physical component health-related quality of life scores. Anxiety and depression were assessed using the Hospital Anxiety and Depression Scale. Perceived stress was measured with the perceived stress scale. Health-related quality of life was measured using the SF-36 (36-Item Short-From Survey). The SF-36 examines eight dimensions: physical functioning (10 items); role limitations due to physical health problems (four items); bodily pain (two items); general health (five items); vitality (four items); social functioning (two items); role limitations due to personal or emotional problems (three items); mental health (five items); and perceived change in health (one item).

Smoking status was ascertained using the Ottawa Model for Smoking Cessation assessment tool^11^; moderate-to-vigorous physical activity (metabolic equivalent of task-minutes per week, minutes per week, 150 minutes per week or longer) was reported using the International Physical Activity Questionnaire; whether individuals met fruit and vegetable intake recommendations was measured with the Rapid Eating and Activity Assessment for patients; and medication adherence was ascertained using a validated assessment tool.

Sociodemographic characteristics known to be associated with postpartum and cardiovascular health outcomes such as age, marital status, education, employment status, race and household income were self-reported during the baseline visit. Data on medical characteristics, including obstetric history and any family history of CVD, were extracted from participants' medical charts, if available, or were obtained from participants during the baseline visit.

All statistical analyses were performed with Stata/SE 15.1. Sociodemographic and clinical characteristics were presented as either mean±SD or frequency (%). Participants were included in the analyses if they had adequate information to generate a Lifetime Risk Score and Framingham Risk Score at 6 and 12 months follow-up, respectively. Little's Missing Completely at Random test was used to assess whether data were missing at random or if there was a pattern to their absence. In a sensitivity analysis, we compared baseline variables between participants who were lost to follow-up and those who completed the study. We performed repeated measures analysis of variance and Cochran Q tests (extension of McNemar test) to compare baseline measures with outcomes at 6 months and 12 months follow-up for continuous and categorical variables, respectively. All statistical significances were two sided and accepted at *P*<.05.

## RESULTS

**Fig. 2.**
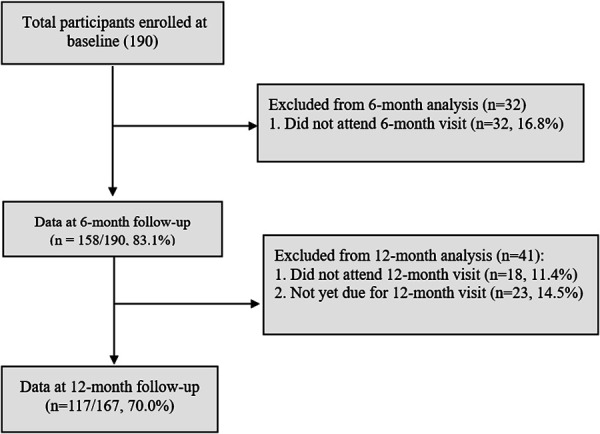
Flow chart of study participants.

Of the 190 participants with a history of HDP who were enrolled in the CP-PP at baseline, 83.1% (158/190) completed the 6-month follow-up; 32 women were lost to follow-up (Fig. 2). Of the 190 participants, 117 (70.0%) completed the 12-month follow-up at the time of this analysis (18 women were lost to follow-up), and 23 were not due for the 12-month follow-up visit.

*Quansah. Effectiveness of Postpartum Program on CVD Risk Scores. O&G Open 2025*.

Table 1 describes the baseline sociodemographic and health characteristics of participants. Participants were, on average, 12 months postpartum. The mean age, weight, and BMI at baseline were 36.6±4.8 years, 81.2±19.9 kg, and 30.5±6.7, respectively. The majority of the participants were married (82.3%), had university-level education (87.9%), and were full-time employees (70.3%). A quarter (24.2%) had a known family history of CVD. We found no differences in baseline variables between participants who completed the study and those who were lost to follow-up.

**Table 1.** Baseline Sociodemographic and Health Characteristics of the Participants (N=190)

Variable	Value
Age (y)	36.6±4.8
Marital status (n=175)	
Single	10 (5.7)
Married	144 (82.3)
Common law	17 (9.7)
Divorced or separated	4 (2.3)
Missing	15 (7.9)
Education (n=141)	
Elementary	1 (0.71)
High school	3 (2.1)
College or trade certificate	13 (9.2)
University	124 (87.9)
Missing	49 (25.8)
Employment status (n=175)	
Full-time	123 (70.3)
Part-time	12 (6.9)
Self-employed	13 (7.4)
Unemployed	3 (1.7)
Retired	4 (2.3)
Other	20 (11.4)
Missing	15 (7.9)
Race (n=174)	
Asian	14 (8.1)
Black	2 (1.2)
White	158 (90.8)
Missing	16 (8.4)
Household income ($) (n=164)	
Less than 10,000	3 (1.7)
10,000–25,000	11 (6.3)
25,001–35,000	4 (2.3)
35,001–50,000	13 (7.4)
50,001–75,000	23 (13.1)
More than 75,000	110 (62.9)
Missing	26 (13.7)
Family history of CVD	46 (24.2)
Smoking status, yes	31 (16.3)
Gravida (n=153)	
1	50 (32.7)
2	53 (34.6)
3 or more	50 (32.7)
Missing	37 (19.5)

CVD, cardiovascular disease.

Data are mean±SD or n (%).

Table 2 summarizes the changes in global CVD risk and clinical, psychosocial, and health behavior outcomes of all enrolled participants at each visit during the 12-month program. Compared with baseline, there was an 11.4% absolute reduction in Lifetime Risk score at 6-month follow-up and 22.6% reduction at 12-month follow-up; Framingham Risk Score reduced by 33.7% at 6-month follow-up and 53.7% at 12-month follow-up (all *P*≤.001).

**Table 2.** Changes in Clinical Characteristics After the 12-Month Intervention Program Among CardioPrevent Postpartum Program Participants

Variable	Baseline (N=190)	6 mo (n=158)	12 mo (n=117)	*P* *			
				Overall	Baseline vs 6 mo	Baseline vs 12 mo	6 mo vs 12 mo
Global CVD risk							
Lifetime Risk Score (%)	32.3±9.9	28.6±12.2	25.0±12.1	**<.001**	**.014**	**<.001**	.560
Framingham Risk Score (%)	35.0±12.7	23.2±11.1	16.2±10.8	**<.001**	**.001**	**<.001**	**<.001**
Clinical outcomes							
Weight (kg)	81.7±19.4	79.6±19.3	74.1±19.8	**.001**	**.003**	**<.001**	**.002**
BMI (kg/m^2^)	30.5±6.7	29.6±6.8	27.3±7.3	.612	.675	**.021**	**.031**
Waist circumference (cm)	96.9±15.5	92.1±13.3	89.5±14.9	**.010**	**.020**	**<.001**	.007
FPG (mmol/L)	4.9±0.9	5.0±0.6	4.9±0.6	.646	.651	.821	.721
Hb A_1c_ (%)	5.41±0.50	5.40±0.38	5.43±0.37	.853	.451	.236	.621
Triglycerides (mmol/L)	1.43±1.01	1.15±0.73	1.1±0.72	**<.001**	**<.001**	**<.001**	.606
HDL (mmol/L)	1.4±0.46	1.37±0.34	1.4±0.34	**<.001**	**<.001**	**<.001**	.890
LDL (mmol/L)	2.9±0.8	2.75±0.78	2.6±0.7	**<.001**	**.004**	**<.001**	.050
TC (mmol/L)	5.0±0.9	4.66±0.84	4.5±0.82	**<.001**	**<.001**	**<.001**	.072
TC/HDL ratio	3.65±1.13	3.55±1.01	3.38±0.89	.094	.841	.090	.627
Metabolic syndrome							
Waist circumference, defined	102 (53.6)	83 (52.5)	51 (43.5)	**.004**	.183	**<.001**	.097
BMI, defined	97 (51.0)	79 (50.0)	49 (41.8)	**.002**	.079	**<.001**	**.001**
Psychosocial outcomes							
QOL mental component score	43.54±9.9	46.9±9.9	48.0±8.7	**.003**	**.002**	**.001**	.824
QOL physical component score	48.4±8.4	56.6±75.2	50.5±8.8	.173	.194	.231	.731
Postpartum depression score	6.4±3.5	4.5±3.0	3.8±3.15	**<.001**	**<.001**	**<.001**	.186
Anxiety score	7.1±3.4	5.9±4.6	5.1±3.4	**<.001**	**.006**	**<.001**	.216
Perceived stress score	21.2±3.9	10.6±10.3	6.8±9.4	**<.001**	**<.001**	**<.001**	**<.001**
Health behavior outcomes							
Current daily cigarette smoking, yes†	31 (16.3)	3 (1.9)	2 (1.7)	**<.001**	**<.001**	**<.001**	**<.001**
Weekly MVPA minutes	111.5±128.0	163.2±178.2	142.4±116	**.003**	**.003**	.213	.771
Meeting MVPA guidelines†	92 (48.4)	104 (65.8)	88 (75.21)	**<.001**	**<.001**	**<.001**	**<.001**
Meeting fruit and vegetable recommendations†	41 (21.5)	84 (53.2)	52 (44.44)	**<.001**	**<.001**	**<.001**	**<.001**
Medication adherence, yes†	56 (29.4)	50 (31.6)	57 (48.71)	**<.001**	**<.001**	**<.001**	**<.001**

CVD, cardiovascular disease; BMI, body mass index; FPG, fasting plasma glucose; Hb A_1c_, hemoglobin A_1c_; HDL, high-density lipoprotein; LDL, low-density lipoprotein; TC, total cholesterol; QOL, quality of life; MVPA, moderate-to-vigorous physical activity.

Data are mean±SD or n (%) unless otherwise specified.

Bold indicates statistical significance (*P*< .05).

* *P*-values derived from repeated measures analysis of variance (continuous variables) or McNemar test (categorical variables) unless otherwise specified.

† Cochran Q test (extension of McNemar test).

Among participants who completed the 12-month follow-up visit, significant decreases in weight (81.7±19.4 vs 74.1±19.8 kg, *P*<.001), waist circumference (96.3±15.5 vs 89.5±14.9 cm, *P*<.001), and BMI (30.5±6.7 vs 27.3±7.3, *P*=.031) were observed between baseline and 12-month follow-up, respectively, but FPG and hemoglobin A_1c_ levels were similar. Significant reductions were observed for triglycerides, LDL cholesterol, and total cholesterol levels between baseline and 12 months follow-up; however, HDL cholesterol also decreased at 12 months, and the total cholesterol/HDL ratio remained similar. Compared with the 50.0% (95/190) of participants who had hypertension at baseline, only 4.3% (5/117) of those who completed the study had hypertension at the 12-month follow-up. These differences led to significant reductions in the prevalence of metabolic syndrome (metabolic syndrome based on waist circumference: 53.6% vs 43.5%; metabolic syndrome based on BMI: 51% vs 41.8%) at end of the program.

The intervention was associated with improvement in the rates of postpartum depression, anxiety, and perceived stress scores at 12 months compared with baseline. Specifically, we observed a 40.6% absolute reduction in postpartum depression, a 28.2% absolute reduction in anxiety, and a 67.9% absolute reduction in perceived stress at 12 months compared with baseline. We observed significant improvements in health-related quality of life mental component scores between the baseline and 12-month follow-up; however, there was no change in physical component scores. There was an absolute decrease of 93.5% in current, self-reported daily smoking rates between baseline and 12-month follow-up (*P*<.001). When comparing baseline with 12-month outcomes, the proportion of women meeting moderate-to-vigorous physical activity guidelines increased (48.4% vs 75.2%, *P*<.001), and medication adherence improved (29.4% vs 48.7%, *P*<.001). The proportion of women who met guideline-recommended fruit and vegetable intake increased by an absolute 26% (21.5% vs 44.4%, *P*<.001).

## DISCUSSION

In this cohort of women with previous HDP, we observed important associations between implementation of a 12-month technology-enabled behavior-change coaching program and reductions in CVD risk factors. Importantly, there were pronounced reductions in both Lifetime Risk Score and Framingham Risk Score, and in clinical outcomes such as body weight, waist circumference, triglycerides, LDL cholesterol, total cholesterol, blood pressure, and rates of metabolic syndrome. Psychosocial outcomes such as depression, anxiety, and perceived stress significantly improved, as did health-behavior outcomes such as daily smoking, physical activity, fruit and vegetable intake, and medication adherence. We observed that outcomes were either maintained or improved at 6 months or 12 months when compared with baseline.

The observed reductions in global risk scores and clinical outcomes corroborate the results of an earlier evaluation of the CardioPrevent program, which observed a 16.6% reduction in Framingham Risk Score and reductions in total cholesterol, blood pressure, smoking, and BMI.^8^ Notably, this previous study observed greater reductions in Framingham Risk Score among female participants compared with males. The absolute reductions in global risk scores observed in our study of women with previous HDP demonstrate that the intervention was not only associated with improvements, but also highlights the early benefits of interdisciplinary lifestyle and behavior-change interventions for improving CVD risk factors in the postpartum period.^12^ Although one small (n=31) randomized clinical trial of a web-based behavioral intervention program that involved women with a history of preeclampsia found no reduction in absolute CVD risk measured by Framingham Risk Score between baseline and 3 months’ postpartum,^13^ our results are consistent with the results of a postpartum cardiovascular risk-reduction program among women with recent preeclampsia that led to improvements in weight and BMI at 6-month follow-up^14^ and with those of a telehealth program that led to weight and BMI reductions at 12 months postpartum.^15^ Furthermore, our findings are comparable with a study in which a 12-week postpartum lifestyle intervention that included exercise training for women with previous preeclampsia led to significant reduction in most components of metabolic syndrome such as BMI, waist circumference, total cholesterol, and LDL.^16^

Compared with the PE-NET (Preeclampsia New Emerging Team) cohort in Ontario, Canada, which included women with preeclampsia who did not receive a postpartum risk-reduction intervention,^17^ our study observed an absolute 18% decrease in the prevalence of metabolic syndrome based on BMI at approximately 1 year postpartum (from 51% to 42%), whereas the PE-NET cohort with no intervention observed an increase in metabolic syndrome prevalence of 11% (from 18% to 29%) over 1 year. Given the numerous cardiovascular consequences of metabolic syndrome, these differences highlight the importance of early CVD risk-reduction interventions.

The CP-PP intervention was also associated with clinically meaningful improvements in lifestyle and behavioral risk factors for CVD^18,19^; previous studies have shown that behavior modifications can significantly reduce the incidence of CVD (by up to 80%).^6^ Although an estimated 50% of women who smoke before pregnancy quit smoking before or during pregnancy, among those who quit, nearly half relapse to smoking during the postpartum period.^20^ In contrast, we observed a highly significant 94% absolute decrease in daily smoking rates between baseline and end of intervention. Increases in the proportions of women meeting moderate-to-vigorous physical activity guidelines and medication intake adherence also were observed. Despite the general difficulty in influencing fruit and vegetable intake, our program led to a 26% increase in the proportion of women who met guideline-recommended fruit and vegetable intake.^21^ Improvements were also observed in postpartum depression, anxiety, perceived stress, and quality of life mental components scores at the end of the program. These results are consistent with those of a behavior-change intervention study involving women who experienced preeclampsia, which led to improved self-efficacy in achieving a healthy diet and reducing physical inactivity, and results of a recent postpartum telehealth-supported lifestyle intervention that led to improvement in depressive symptoms in postpartum women.^22^ As per a recent systematic review, the observed decreases in postpartum weight and BMI in our study could be associated with the significant reductions in postpartum stress, depression, and anxiety.^23^

Multiple health behavior–change interventions can be an effective CVD risk factor reduction program for women with previous HDP.^24^ However, factors including low awareness of CVD risk, confusing messages in the media, and home and caregiving responsibilities such as childcare necessities^25^ are barriers to accessing care in the postpartum period. In addition, perceived lack of time, lack of structured patient counseling, and fragmentation of care are health care professional–associated barriers that prevent women from accessing postpartum CVD care services.^26^ Technology-enabled programs such as the CP-PP could be effective approaches to improving follow-up rates and adherence during the postpartum period. The use of virtual programs not only increases uptake of interventions, but also provides the opportunity to tailor interventions to the individual based on specific risk factors and health goals.

This study did not include a control group; therefore, observed reductions in CVD risk factors may not be attributed to the intervention program, alone. It could be argued that certain outcomes would have improved on their own over time in these postpartum women; however, this is less likely because our participants enrolled at, on average, 1 year postpartum. Although we observed important decreases for weight and BMI, for example, others have demonstrated that between 20% and 50% of women retain a substantial portion of the weight gained during pregnancy when measured at 6 months to 2 years postpartum.^27–29^ We also observed a substantial decrease in metabolic syndrome prevalence, compared with the PE-NET cohort, which observed an increase in metabolic syndrome among women with prior HDP who received no intervention.^17^ Although 26.3% (50/190) were lost to follow-up at either 6 months (n=32) or 12 months (n=18), a sensitivity analysis found no differences in baseline characteristics and outcomes between those who completed the study and those who were lost to follow-up. The follow-up period of 12 months in our study is longer than several other risk-reduction programs; however, an extended period of follow-up would be beneficial in assessing whether intervention effects are sustained. The psychosocial and behavioral outcomes were self-reported using validated questionnaires but could be subject to reporting bias; future studies would benefit from objective measures, when appropriate. Finally, although program participants were representative of reproductive-aged women in the Ottawa region in terms of rates of postsecondary education and marital status, they were not in terms of race. In Ottawa, Ontario more than 11% of women between the ages of 25 and 54 are of Asian descent and more than 7% of African or Caribbean descent, compared with 8.1% and 1.2% among our study participants, respectively.^30^ Future studies should evaluate the acceptability and effectiveness of postpartum behavior-change interventions on population subgroups (eg, race, socioeconomic status, geographic location).

The CP-PP led to significant and clinically meaningful reductions in global CVD risk and clinical, psychosocial, and behavioural outcomes at 6-months follow-up, and these improvements were mostly maintained at 12-months in a population of women with a recent history of HDP. Multidisciplinary health-behaviour technology-enabled interventions can be used successfully to reduce face-to-face appointments, increase patient interactions remotely, and reduce CVD risk. The CP-PP is scalable and can be disseminated to improve preventive care within this high-risk population.
